# Familial Cancer Variant Prioritization Pipeline version 2 (FCVPPv2) applied to a papillary thyroid cancer family

**DOI:** 10.1038/s41598-018-29952-z

**Published:** 2018-08-02

**Authors:** Abhishek Kumar, Obul Reddy Bandapalli, Nagarajan Paramasivam, Sara Giangiobbe, Chiara Diquigiovanni, Elena Bonora, Roland Eils, Matthias Schlesner, Kari Hemminki, Asta Försti

**Affiliations:** 1 Division of Molecular Genetic Epidemiology, German Cancer Research Center (DKFZ), D69120 Heidelberg, Germany; 2 Division of Theoretical Bioinformatics, German Cancer Research Center (DKFZ), D69120 Heidelberg, Germany; 3 Medical Faculty Heidelberg, Heidelberg University, D69120 Heidelberg, Germany; 4 Unit of Medical Genetics, S.Orsola-Malpighi Hospital, 40138 Bologna, Italy; 5 Department of Bioinformatics and Functional Genomics, Institute of Pharmacy and Molecular Biotechnology (IPMB) and BioQuant, Heidelberg University, D69120 Heidelberg, Germany; 6 Bioinformatics and Omics Data Analytics, German Cancer Research Center (DKFZ), D69120 Heidelberg, Germany; 7 Center for Primary Health Care Research, Lund University, Malmö, Sweden

## Abstract

Whole-genome sequencing methods in familial cancer are useful to unravel rare clinically important cancer predisposing variants. Here, we present improvements in our pedigree-based familial cancer variant prioritization pipeline referred as FCVPPv2, including 12 tools for evaluating deleteriousness and 5 intolerance scores for missense variants. This pipeline is also capable of assessing non-coding regions by combining FANTOM5 data with sets of tools like Bedtools, ChromHMM, Miranda, SNPnexus and Targetscan. We tested this pipeline in a family with history of a papillary thyroid cancer. Only one variant causing an amino acid change G573R (dbSNP ID rs145736623, NM_019609.4:exon11:c.G1717A:p.G573R) in the carboxypeptidase gene *CPXM1* survived our pipeline. This variant is located in a highly conserved region across vertebrates in the peptidase_M14 domain (Pfam ID PF00246). The *CPXM1* gene may be involved in adipogenesis and extracellular matrix remodelling and it has been suggested to be a tumour suppressor in breast cancer. However, the presence of the variant in the ExAC database suggests it to be a rare polymorphism or a low-penetrance risk allele. Overall, our pipeline is a comprehensive approach for prediction of predisposing variants for high-risk cancer families, for which a functional characterization is a crucial step to confirm their role in cancer predisposition.

## Introduction

Oncogenomics has been boosted with rapid advancements in the next-generation sequencing (NGS) technologies in the last 10 years with large consortia describing several thousands of somatic variants. However, there has been far less success in the discovery of new cancer predisposing genes (CPGs) as only some new genes were identified using germline genome sequencing^1^. The major fraction of CPGs were discovered in the late 1990s using the familial linkage analysis^1^. Since then the interest in collecting cancer pedigrees diminished and the consequence has been that whole exome/genome sequencing (WES/WGS)-based family studies have been forced to resort either to small pedigrees or single cases from affected families^2^. In cancer studies both types of approaches have been used^3–6^. CPGs found include for example an *NTHL1* variant in colorectal cancer^3^ and an *RECQL* variant in breast cancer^4^.

As pedigree-based studies have a high discriminatory power if samples from many affected and unaffected members are available, we recently introduced the familial cancer variant prioritization pipeline (FCVPP^5^), which is a pipeline capable of detecting rare germline variants and their corresponding CPGs. In the current study, we describe FCVPPv2, an upgraded version of our FCVPP pipeline^5^. FCVPPv2 prioritizes rare deleterious and regulatory germline variants, both in the coding and non-coding region for cancer families. The advantages of this approach are several-fold such as (a) reducing the large number of variants through the pedigree segregation step; (b) assessing the deleterious nature of missense variants by a combination of 12 ranking tools and 5 intolerance scores; (c) analysis of non-coding variants by specialized tools such as Miranda^7^ and Targetscan 7.0^8^ for 3′ UTR variants, SNPnexus^9^ for 5′ UTR variants and FANTOM5 and SNPnexus^9^ for variants in enhancers^10^ and promoters^11,12^ and (d) this pipeline also takes advantages of the improvements of population frequencies in public databases, which assists inferring rarity of a particular variant. Herein, we present the improvements of FCVPPv2 as well as its application to a pedigree of papillary thyroid cancer (PTC).

## Results

### Familial cancer variant prioritization pipeline version 2 (FCVPPv2) comes with several improvements

**Figure 1:**
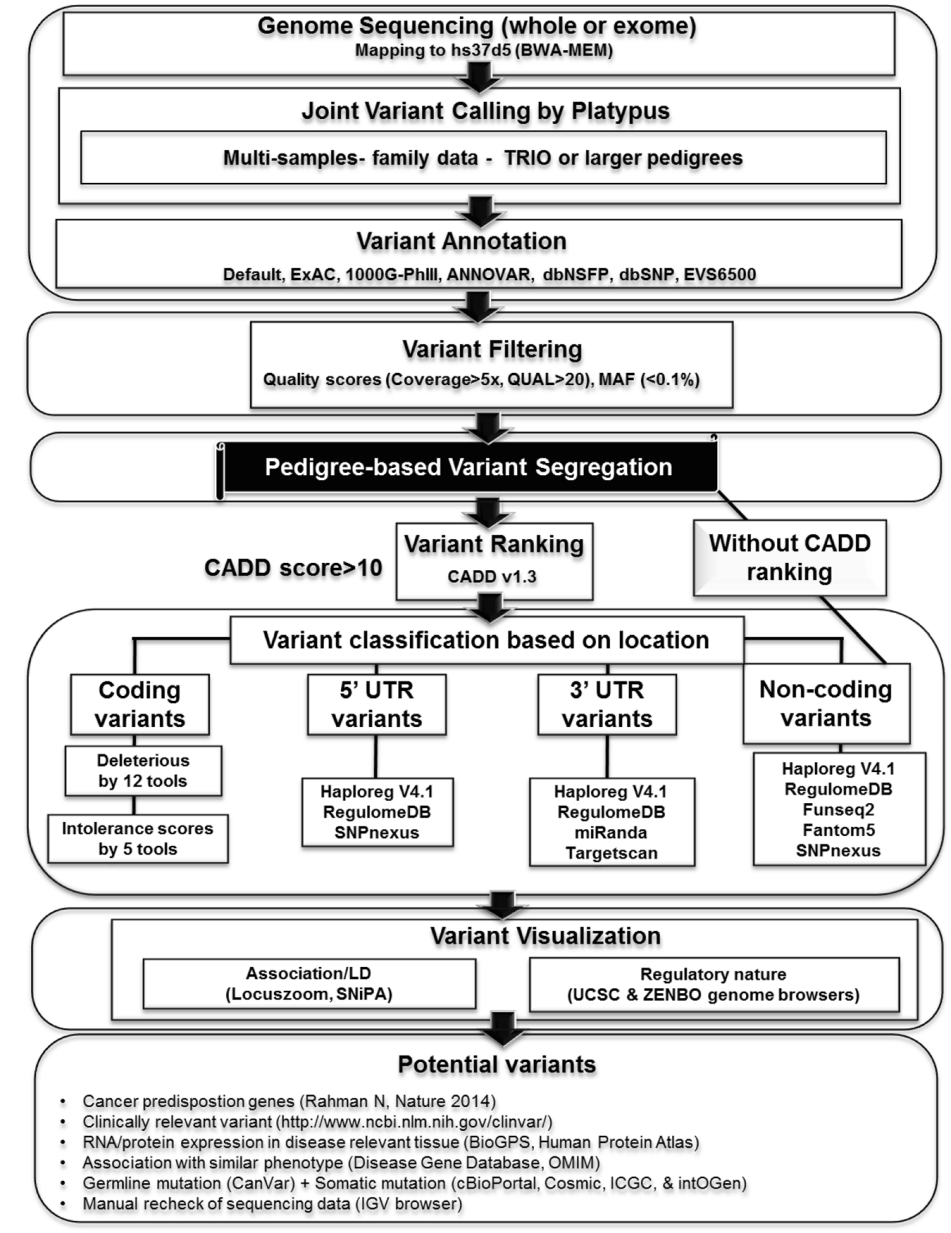
Summary of familial cancer variant prioritization pipeline version 2 (FCVPPv2). This pipeline uses platypus tool^13^ for joint variant calling after mapping of the sequencing reads from cases and controls. FCVPPv2 uses several external tools for variant annotation namely ExAC, 1000 Genomes phase III data, ANNOVAR and dbNSFPv3, dbSNP and EVS6500. For candidate variants the variants are filtered using read quality parameters like coverage and quality scores (QUAL) must be >5 and >20, respectively. Minor allele frequency (MAF) must be below 0.1% in the European populations in all used databases. Furthermore, these variants are screened with respective to family-pedigree and this is the most critical step in the germline genomics (shown in black shade). After this step, variants are ranked with the help of CADD v1.3^20^ and any variants with CADD PHRED score of >10 belongs to top 10% for probable functional and deleterious variants in the human genome. These deleterious variants are subsequently divided into 4 different categories based on their locations. The coding variants are considered deleterious based on the consensus from 12 deleteriousness prediction tools and 5 intolerance scores. Variants in the 5′ UTR are considered regulatory based on the Haploreg V4.1^21^, RegulomeDB^22^ and SNPnexus^9^ while variants in the 3′ UTR are regulatory if supported by the presence of miRNA binding site using Miranda^7^ and Targetscan 7.0^8^ tools and additional hints are received from Haploreg V4.1^21^ and RegulomeDB^22^. For variants in the non-coding segments we combined several state-of- the-art tools such as chromHMM, Segway, FunSeq2 and FANTOM5 data. Non-coding (intergenic and intronic) variants may not always have CADD > 10 even though they will have regulatory implications, so we analyzed all non-coding variants after pedigree segmentation, either with or without CADD > 10. Putative deleterious or regulatory variants are visualized using Locuszoom, SniPA and UCSC genome browser. Potential variants are also checked with sets of additional features, e.g. list of known CPGs^1^ and clinically relevant variants (ClinVar), expression data and somatic mutations. We also checked the sequencing data of all cases and controls in a particular family for correctness using the IGV browser.

**Figure 2:**
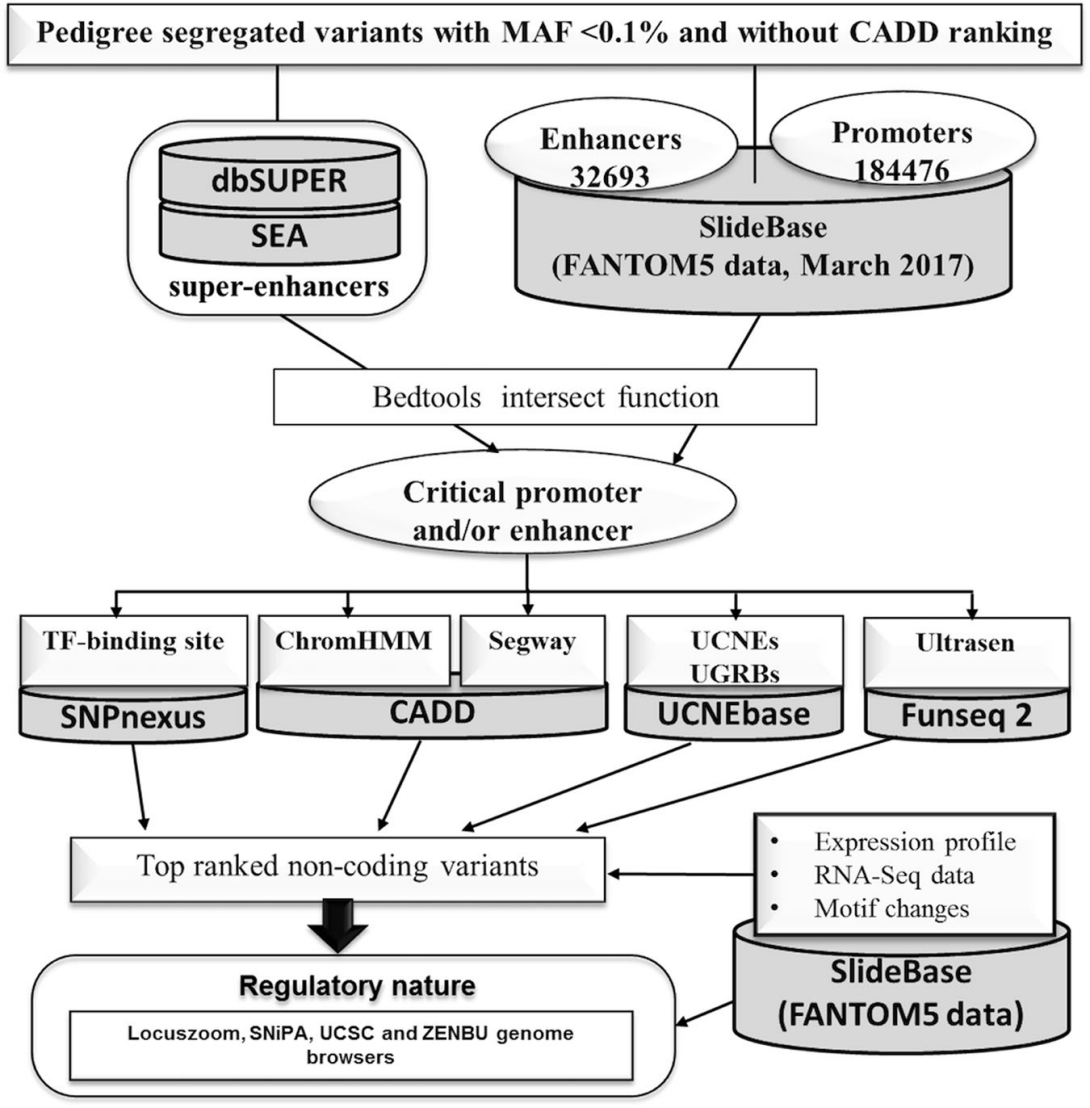
Overview of strategies for regulatory variant detection in the non-coding segments of the human genome. We utilized the FANTOM5 data using the SlideBase Tool (*slidebase.binf.ku.dk*) with 32693 enhancers and 184476 promoters (downloaded in March 2017). We matched our variants (pedigree segregated) with FANTOM5 data using Bedtools intersect to retrieve a list of potentially critical variants localized within promoters and/or enhancers, and we examined the status of transcription factor (TF) binding sites using SNPnexus^9^. We checked the signals for chromatin binding using ChromHMM and genomic segmentation data from Segway via CADDv1.3^20^. In addition, we examined if the putative noncoding regulatory variants were localized in the ultra-conserved non-coding elements (UCNEs) and their clusters, also known as ultra-conserved genomic regulatory blocks (UGRBs) with the help of UCNEbase^24^ and also if these variants were located in ultra-sensitive and sensitive regions (Ultrasen), defined by FunSeq2^23^. The top-ranked variants were examined for their regulatory nature by using Locuszoom, SniPA, UCSC and ZENBU genome browsers. We also examined if the putative enhancer variant fall into the category of super-enhancers using super-enhancer archive (SEA)^25^ and dbSUPER^26^. Expression profile, RNA-seq data-based information and motif changes and disruptions were gathered with help from FANTOM5 data via the SlideBase.

Overview of FCVPPv2 is provided in Figs 1 and 2 and description of this work involves several abbreviations of scientific words and tools as summarized in Supplementary Table S1. FCVPPv2 has implemented platypus tool^13^ for joint variant calling. It combines several resources for variant annotation like ANNOVAR^14^, exome aggregation consortium (ExAC^15^), exonic variant server with 6500 samples (EVS6500^16^), 1000 Genomes phase III^17^, dbNSFP v3.0^18^, and dbSNP^19^.

Variants are filtered with criteria using read quality parameters like coverage and quality scores (QUAL) must be >5 and >20, respectively. FCVPPv2 uses minor allele frequency (MAF) below 0.1% in the European populations in all used databases. The hallmark of FCVPPv2 is use of family-pedigree variant screening (Fig. 1). Post this step, CADD v1.3^20^ based variant filtering is performed with PHRED CADD score of 10 as a cut-off. Subsequently, deleterious variants are segregated on their locations. The coding variants are considered deleterious based on the consensus from 5 intolerance scores, 3 conservational scores and 12 deleteriousness prediction tools (Fig. 1, Tables 1 and 2). FCVPPv2 characterizes the regulatory nature in the UTR regions by combining a set of tools like the Haploreg V4.1^21^, RegulomeDB^22^ and SNPnexus^9^ for variants in 5′ UTR and miRNA binding sites using Miranda^7^ and Targetscan 7.0^8^ tools for variants in 3′ UTRs (Fig. 1).

**Table 1:** Summary of intolerance scores and conservational scores.

Tools	Details	Score Range	Significant score	Ref.
Residual Variation Intolerance Score (RVIS) RVIS - NHLBI-ESP6500 data set	based upon allele frequency data	Negative to Positive	RVIS < 0 - intolerant RVIS > 0 - tolerant	^60^
RVIS - ExAC data set				
RVIS - local data set				
pLI score	Developed by ExAC Consortium for Loss-of-Function (LoF) mutations		pLI ≥ 0.9 - highly LoF-intolerant pLI ≤ 0.1 - LoF tolerant	^15^
Z-score	Developed by ExAC Consortium for missense and synonymous variants		Positive Z scores –intolerant Negative Z scores -tolerant	^15^
Genomic Evolutionary Rate Profiling (GERP)		−12.3 to 6.17	>2	^61^
PhastCons		0 to 1	>0.3	^62^
Phylogenetic P-value (PhyloP)		−14 to +6	≥3.0	^63^

**Table 2:** Summary of used tools for deleteriousness prediction for missense variants.

Tools	Methodology	Score ranges	Prediction	References
Sorting Intolerant from Tolerant (SIFT)	Position-specific scoring matrix (PSSM) with Dirichlet priors Sequence based. uses PSI-BLAST	0 to 1*	D – Damaging (<0.05) T – Tolerated (>0.05)	^64^
Polymorphism Phenotyping version-2 (PolyPhen-v2)	Naïve Bayes classifier trained using supervised machine-learning Sequence and structure based	0 to 1**	D – probably damaging (0.957–1) P – possibly damaging (0.453–0.956) B – benign (0.00–0.452)	^65^
PolyPhen2_HDIV (HumDiv^$^)				
Polyphen2_HVAR (HumVar^%^)				
Log ratio test (LRT)	Uses log ratio test Sequence based	0 to 1***	D – Deleterious N – Neutral U – Unknown	^69^
MutationTaster	Naïve bayes model operated on the integrated data source Based on sequence and annotation.	0 to 1**	A– disease_causing_automatic D – disease_causing (>0.5) N – polymorphism (<0.5) P – polymorphism_automatic	^70^
MutationAssessor	Multiple sequence alignment (MSA) and conservation scores	−5.135 to 6.49**	H – High L – Low M – Medium N – Neutral	^71^
Functional Analysis Through Hidden Markov Models (FATHMM)	Hidden Markov models (HMM) Based on sequences and protein domains	−18.09 to 11.0*	D – Damaging (< = −1.5) T– Tolerated (>−1.5)	^72^
MetaSVM	Support vector machine (SVM) based score, derived by incorporating different scores^#^	−2 to 3**	D – Damaging (>0) T– Tolerated (<0)	^18^
MetaLR	Logistic regression (LR) based score, derived by combining different scores^#^	0 to 1**	D – Damaging (>0.5) T – Tolerated (<0.5)	^18^
Variant Effect Scoring Tool version 3 (VEST3)	Supervised machine learning-based method Combines conservational and structural features	0 to 1**	NA	^73^
Protein Variation Effect Analyzer (PROVEAN)	Pair-wise alignment-based scoring method	−14 to 14*	D – Damaging (< = −2.5) N– Neutral (>−2.5)	^74^
Reliability index (RI)	SVM based Combines protein sequence and structural features	0 to 10**	D – Damaging (≥5) N– Neutral (<5)	^75^

^*^Lower scores indicate deleterious nature.

**Higher scores indicate deleterious nature.

***Score cannot decide deleterious nature.

^$^HumDiv - collection of mendelian disease variants (5564 deleterious + 7539 neutral in 978 human protein) against divergence from close mammalian homologs of human proteins (> = 95% sequence identity).

^%^HumVar - compilation of all human variants (22196 deleterious + 21119 neutral) associated with some disease (non-cancer mutations) or loss of activity/function vs. common (MAF > 1%) human polymorphism with no reported association with a disease.

^#^10 scores from SIFT, PolyPhen-2 HDIV, PolyPhen-2 HVAR, GERP++, MutationTaster, Mutation Assessor, FATHMM, LRT, SiPhy and PhyloP and the maximum frequency observed in the 1000 G data.

Above 98% of the human genome is non-coding and dealing with non-coding variants is a daunting task. No tools can accurately predict the regulatory nature of non-coding variants. To overcome this issue, FCVPPv2 uses a combination of the several standard tools like chromHMM, Segway, FunSeq2^23^ and FANTOM5 data (Fig. 2). FCVPPv2 focuses the FANTOM5 data by matching variants within promoters and/or enhancers using Bedtools intersect function. FCVPPv2 makes assessments of transcription factor (TF) binding sites using SNPnexus^9^. This pipeline makes use of signals for chromatin binding using ChromHMM and genomic segmentation data from Segway via CADDv1.3. Additionally, FCVPPv2 checks if a putative non-coding regulatory variant is localized in the ultra-conserved non-coding elements (UCNEs) or ultra-conserved genomic regulatory blocks (UGRBs) with the help of UCNEbase^24^, and in ultra-sensitive and sensitive regions (Ultrasen), defined by FunSeq2^23^. FCVPPv2 uses Bedtools intersect function to assign variants in regions of super-enhancers using super-enhancer archive (SEA)^25^ and dbSUPER^26^ databases.

FCVPPv2 visualises top ranked variants for their regulatory natures using different genome browsers like Locuszoom^27^, SNiPA^28^, the UCSC^29^ and ZENBU^30^ (Fig. 1). The status of RNA and protein expression for genes carrying potential deleterious variants is examined with the help of FANTOM5, BioGPS^31^ and Human Protein Atlas^32^ (Fig. 1). This tool uses literature mining to check if these variants are found in known lists of cancer predisposing genes (CPGs^1^). Towards end, FCVPPv2 provides a summary of potential variants by combining features from several databases like ClinVar^33^, Online Mendelian Inheritance in Man (OMIM, https://omim.org/), CanVar Browser^34^, cBioPortal^35^, COSMIC^36^, ICGC^37^ and IntOGen^38^ (Fig. 1).

### Application of FCVPPv2 to a papillary thyroid cancer (PTC) family

**Figure 3:**
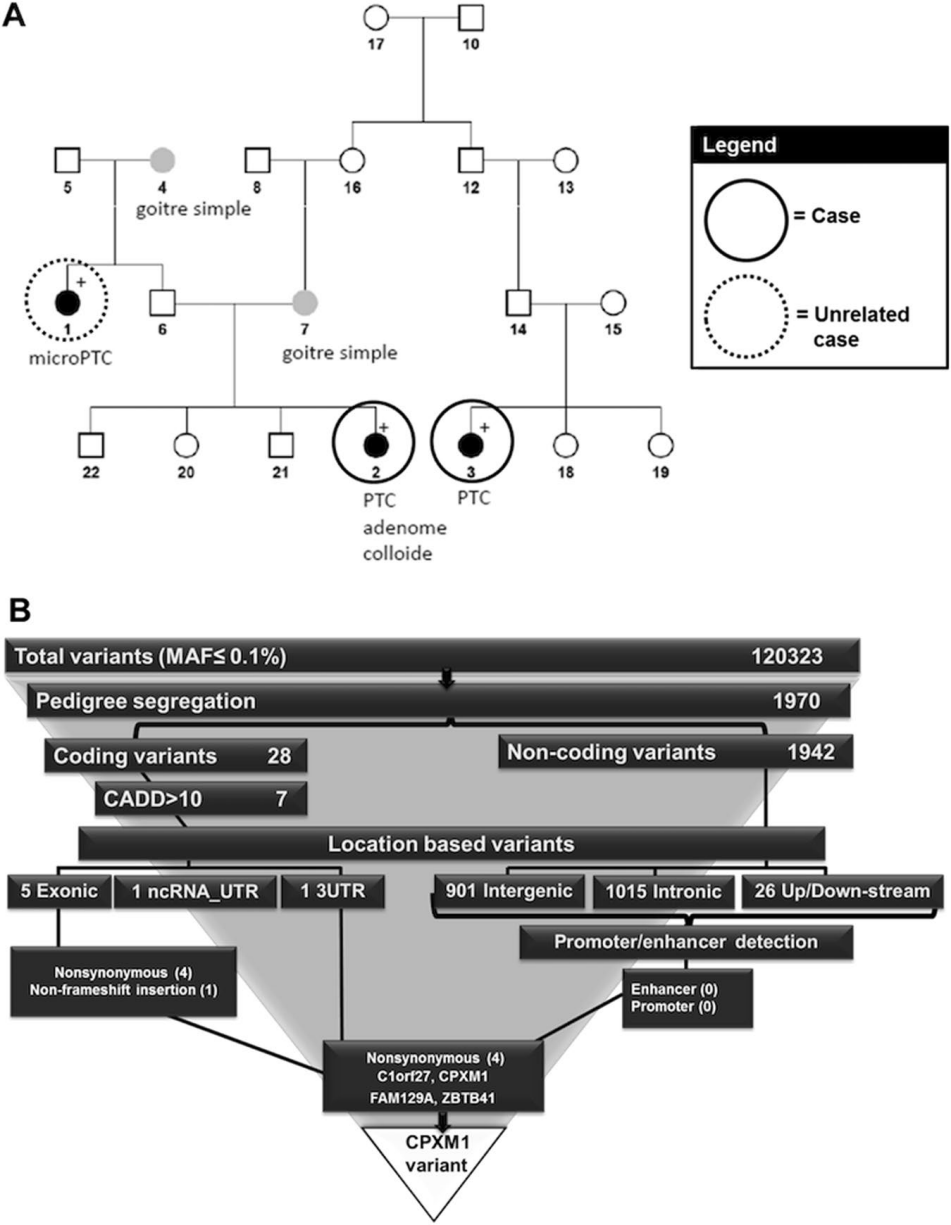
Summary of the papillary thyroid cancer (PTC) family and variant ranking within this family. (**A**) Pedigree of the PTC family. (**B**) Variant ranking for the PTC family and selection of CPXM1 variant as the top deleterious variant.

We used FCVPPv2 to a family with PTC with two distantly related cases (sample no. 2 and 3) and one unrelated case (sample no. 1, Fig. 3A). We ranked top variants for this family after WGS of the three samples. After variant annotation and removal of variants with MAF higher than 0.1% in at least one variant database, a total of 120,323 variants remained (Fig. 3B & Supplementary Table S2). We applied pedigree-filtering criteria imposing that the two related cases must have the variant, while the unrelated case should not have it. With this pedigree filtering approach we narrowed down the potential list of variants to 1970, which is about 1.6% of the initial variant set (Fig. 3B). Subsequently, we examined these variants based on their location in the genome and we found that only 28 of them were located in the coding region, which is 1.4% of all pedigree segregated variants, while 98.6% of them were located in the non-coding region with 1015 intronic, 901 intergenic and 26 up- or down-stream variants (Fig. 3B). Out of the 28 coding variants only 7 had a CADD PHRED score >10 and these included 5 exonic (4 non-synonymous and 1 non-frameshift insertion) and two UTR (1 in 3′ UTR and 1 in ncRNA_UTR) variants (Fig. 3B and Table 3). The non-frameshift insertion and the UTR variants (Table 3) were indels localized in the repetitive regions in the human genome and hence these were not considered further. Of the non-coding variants, none was located within a predicted enhancer or promoter.

**Table 3:** Overview of the 7 top-ranked germline variants detected in the PTC family.

Gene Name	Gene Description	Variant	Variant nomenclature^$^	Variant type	No. of cases	No. of unknown cases	ANNOVAR Annotation	Exonic Classification	CADD score
C1orf27	chromosome 1 open reading frame 27	1_186355211_G_A	NM_017847.5:exon4:c.G326A:p.R109H	SNVs	2	0	exonic	nonsynonymous SNV	25.1
FAM129A	family with sequence similarity 129, member A	1_184792402_T_C	NM_052966.3:exon8:c.A884G:p.K295R	SNVs	2	0	exonic	nonsynonymous SNV	23.9
ZBTB41	zinc finger and BTB domain containing 41	1_197128680_C_T	NM_194314.2:exon10:c.G2539A:p.D847N	SNVs	2	0	exonic	nonsynonymous SNV	23.1
CPXM1	carboxypeptidase X (M14 family), member 1	20_2776248_C_T	NM_019609.4:exon11:c.G1717A:p.G573R	SNVs	2	0	exonic	nonsynonymous SNV	32
KCNE3	potassium voltage-gated channel, Isk-related family, member 3	11_74167200_AATAT_A	NM_005472.4:c.*1097–1097delATAT	Indel	2	0	ncRNA_UTR3	.	11
AR	androgen receptor	X_66765158_T_TGCAGCAGCA	NM_000044.3:c.239_240insGCAGCAGCA	Indel	2	0	exonic	nonframeshift insertion	12.8
NLK	glucose-6-phosphate isomerase	17_26522009_T_TCACA	NM_016231.4:c.*347_*348insCACA	Indel	2	0	UTR3	.	11.7

$ - as per guidelines of the Human Genome Variation Society (HGVS, website http://www.hgvs.org/).

**Figure 4:**
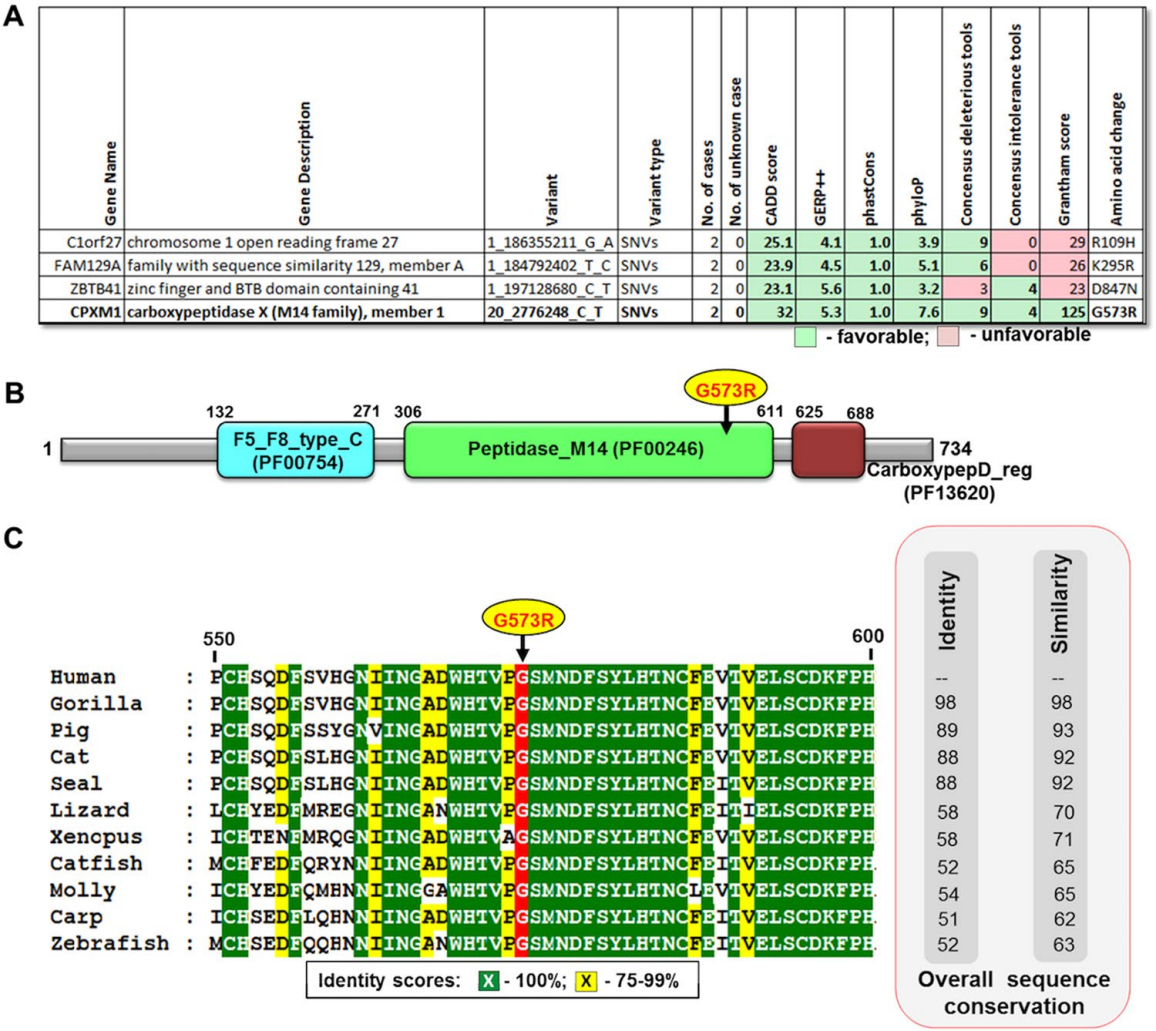
Overview of the top missense variants in the PTC family. (**A**) The 4 top ranked variants are shown with their favorable and unfavorable features. Grantham scores - 0–50 - conservative, 51–100 - moderately conservative, 101–150 - moderately radical and ≥151 - radical. (**B**) Location of the G573R variant in the peptidase M14 domain of CXPM1. (**C**) The G573R variant is localized in a highly conserved region. CXPM1 protein sequences were downloaded from GenBank as human (GenBank ID - NP_062555.1), gorilla (XP_004061758.1), cat (XP_003983774.1), pig (XP_003134381.1), seal (XP_021544821.1), lizard (XP_008120663.1), *Xenopus* (XP_002936314.1), catfish (XP_017320329.1), carp (XP_018934262.1), molly (XP_014844715.1) and zebrafish (XP_693256.4).

We focused on the 4 non-synonymous variants localized in four different genes-*C1orf27* (chromosome 1 open reading frame 27), *CPXM1* (carboxypeptidase X (M14 family), member 1), *FAM129A* (family with sequence similarity 129, member A) and *ZBTB41* (zinc finger and BTB domain containing 41) (Figs 3B and 4A). All these 4 variants had CADD score >20, which indicates top 1% deleterious variants in the human genome. The highest CADD score of 32 was reported for *CPXM1* ranking it to the list of top 0.1% deleterious variants. We prioritized the variant G573R in *CPXM1* as our top candidate, as it had very high conservation scores (GERP++ = 5.3, phastCons = 1.0, and phyloP = 7.6), and 9 out of the 12 deleterious prediction scores and 4 out of the 5 intolerance scores were favoring it. Also amino acid change from glycine to arginine is critical with a Grantham score of 125 (moderately radical, deduced from CADD annotation^20^), while the remaining three variants had a low Grantham score (<30). Additionally, although the three other variants had a CADD score >20, mutations in *C1orf27* and *FAM129A* were classified as tolerated by all 5 intolerance scoring tools (listed in Table 1) and the *ZBTB41* variant was only predicted to be deleterious by 3 out of 12 tools (Fig. 4A). All in all, we found one predicted deleterious missense variant in the *CPXM1* gene, which was not found in any other 77 cancer families (including 4 PTC families), we have whole-genome sequenced by now. We only found two other missense variants in the entire CPXM1 gene in three different families out of the 77 cancer families, each present either in only one case or only one control of the family (Supplementary Table S3). During the course of this study, the NM_019609.4:exon11:c.G1717A:p.G573R variant was identified in one colorectal cancer patient (Supplementary Table S4) as reported in the CanVar Browser^34^ (a database of genetic variants of 1,006 early-onset familial colorectal cancer cases^6^). We also found a rare stop gained variant and 3 more frequent missense variants in this gene in the CanVar Browser^34^ (Supplementary Table S4). Currently, the G573R variant (dbSNP ID - rs145736623) is also listed in the ExAC database with a frequency of 0.0004 in the total population (0.0006 in the European, non-Finnish population). Taken together, as we found the NM_019609.4:exon11:c.G1717A:p.G573R variant in 2 distantly related cases of our PTC family, it may be a low-penetrance allele predisposing to PTC, but it may also be a rare polymorphism.

### Characterization of the *CPXM1* variant and potential roles of the *CPXM1* gene

Our familiar PTC candidate variant (NM_019609.4:exon11:c.G1717A:p.G573R) is located at the end of exon 11 of the *CPXM1* gene, which is composed of 14 exons (Fig. S1A). To confirm the accuracy of variant calling, we examined the genomic data of all sequenced samples using Integrative Genomics Viewer (IGV)^39^, reassuring that this *CPXM1* variant is only present in the two related cases (sample no. 2 and 3, Fig. S1B), but not in the unrelated case (sample no. 1).

The human *CPXM1* gene (also known as *CPX1*) encodes a zinc metallocarboxypeptidase. The human CPXM1 protein is 734 amino acids long (Fig. 4B). Upon Pfam domain scanning, we found that the CPXM1 protein is composed of three Pfam domains, namely F5_F8_type_C (PFam ID - PF00754), peptidase_M14 (PF00246) and carboxypepD_reg (PF13620) located at amino acid positions 132–271, 306–611 and 625–688, respectively. The CPMX1 variant (NM_019609.4:exon11:c.G1717A:p.G573R) is localized in the peptidase_M14 domain (Fig. 4B). Upon examination the 25 amino acids flanking the variant position, we found the G573 residue and the majority of the flanking residues to be highly conserved from human to zebrafish (Fig. 4C). The CPXM1 protein has no carboxypeptidase activity but it is a secreted N-glycoprotein that binds collagen^40^. It has been reported to be involved in adipogenesis through extracellular matrix remodeling^41^. Peroxisome proliferator-activated receptor gamma (PPARγ) is the master adipogenic regulator and it may promote growth and invasion of undifferentiated thyroid cancer (TC) cells^42^. Whether *CPXM1* may predispose to differentiated TC by acting as a complementary regulator to PPARγ in adipogenesis or through extracellular matrix remodeling, remains to be discovered^41^. There is experimental evidence indicating that expression of *CPXM1* is epigenetically regulated in breast cancer and it may act as a tumor suppressor gene^43,44^. Recently, an indirect role for *CPXM1* in PTC was also illustrated, as *CPXM1* was downregulated by a long non-coding RNA (lncRNA, Ensembl ID - ENSG00000273132.1)^45^.

## Discussion

The main theme for understanding the germline cancer genetics is the identification of pathogenic mutations and genes predisposing to cancer. Rapid improvements in scientific and technological aspects of genomics have contributed to revolutionary changes in cancer genetics in particularly in cancer treatment but also in cancer risk assessment, cancer screening and prevention, thus setting up a milestone for approaching towards personalized medicine^46^. With these advancements and decreasing costs, WES/WGS has become the state-of-the-art tool for identifying susceptibility loci in several types of Mendelian diseases^2^. There are a handful of successful reports on disease gene identification for cancer syndromes such as *TERT* promoter mutation^47^ and *POT1* mutations^48^ in familial melanoma, *POLE*, *POLD1*^49^ and *FAN1*^50^ mutations in familial colorectal cancer and *KDR* mutation^51^ in familial Hodgkin lymphoma. However, delivering one out of several millions of human genetic variants as the main cause of hereditary cancer is a daunting computational task^2^. Recently, we developed a pipeline for this purpose, which is known as the FCVPP^5^. We applied several types of improvements to this pipeline while working with different cancer families and now we are representing the second version of this pipeline as FCVPPv2. FCVPPv2 can deal with missense variants in a more sophisticated way by employing 12 deleteriousness assessment tools and 5 intolerance scores. Additionally, it has the capability of dealing with non-coding variants by the use of data from FANTOM5, super-enhancer databases, UCNEbase and FunSeq2, and without CADD filter as CADD may not pick up many non-coding positions as deleterious.

As an example we showed our experience in hunting predisposing genes for PTC in a high risk PTC family. Our approach involved WGS of germline DNA from several affected and unaffected family members. As a consequence, we found that only 1 out of 61 variants (1970 variants out of 120,323 rare variants remained after pedigree segregation) was able to pass the pedigree segregation filter (Fig. 3B). Hence, pedigree-based variant filtering is a highly effective way for filtering out non-causative variants; in this case only <1.6% of the rare variants survived this step.

We identified 28 variants out of 1970 (1.4%) as coding region variants, which is close to the proportion of coding regions of the whole genome (2%). A single coding variant, *CPMX1* (NM_019609.4:exon11:c.G1717A:p.G573R), passed our filtering criteria, CADD score >10, location in a highly conserved region, deleterious in >60% of the prediction tools and intolerance tools predicting it to be deleterious. We had three indel variants in the coding and untranslated regions but they were in repetitive genomic segments and hence were not considered for final prioritization. Furthermore, none of the 1942 non-coding variants were predicted to be located within a promoter or an enhancer. Literature search gave some evidence about a potential function of the *CPXM1* gene in cancer. *CPXM1* may serve as a tumour suppressor in breast cancer^43,44^, potentially through involvement in adipogenesis or extracellular matrix remodelling^40,41^, and it is reported to be down-regulated by a lncRNA in PTC^45^. Our study identified one predicted pathogenic mutation located in the peptidase M14 domain of the CPMX1 protein in the PTC family, yet this domain is inactive in CPMX1 protein and lacks a typical carboxypeptidase function. Recent sequencing data on colorectal cancer and ExAC databases suggest that the identified variant may be a rare polymorphism. Alternatively it may be a low-penetrance PTC predisposing variant, as it was found in two distant relatives, with no other known PTCs in the family. Lack of functional data of the *CPXM1* gene complicates further evaluation of its function and the pathways it is involved. As tumour samples from the mutation carriers of the PTC family are not available, we cannot explore the tumour suppressor nature of *CPMX1* in PTC.

In general, our understanding of human genes and their roles in human diseases, including cancer, are still limited. Lack of proper annotation and unknown physiology hampers mechanistic groundwork for candidate variants. Genes involved in known pathways and with more information in the literature are more likely to be studied further than genes with little or no functional characterization. Without this knowledge or convincing segregation data there may be doubts to accept the detected genes as new bona fide tumor suppressor genes. Recent findings on *HABP2* gene in nonmedullary thyroid cancer^52^, deubiquitinating enzyme coding *BAP1* in multiple cancers^53^, ovarian cancer gene 1 (*OVCA1*)^54^, promyelocytic leukemia protein (*PML*)^55^ and regucalcin (*RGN*)^56^ are offered by variable levels of supporting functional evidence. From these, the *HABP2* mutation^52^ was later shown to be a common polymorphism^57^. Another problem faced by the WES/WGS results in Mendelian diseases is also highlighted in our study: the potentially pathogenic mutation was found only in one family^2^.

An estimate from 2012 suggested that WES/WGS studies in Mendelian diseases have a success rate of about 60–80%^2^. However in cancer only handful of novel CPGs were found through WGS/WES^58^. We think that cancer is more complex than other Mendelian diseases because carcinogenesis is the interplay of germline and somatic events in the form of tumour growth.

In this study, we provide details of a variant prioritization pipeline FCVPPv2 for gene identification in high-risk cancer families applying pedigree segregation-based variant filtering and variant prioritization using state-of-the-art bioinformatics tools and databases. This pipeline detected a predicted deleterious variant in the *CPMX1* gene in a PTC family. However, as recent sequencing efforts have found the variant in both the ExAC population and a colorectal cancer family member, experimental validation of the identified variant and functional characterization of the gene are necessary for mechanistic understanding and evaluation of the potential cancer predisposing nature of CPMX1.

## Material and Methods

### Ethic permissions for the experiments from two committees

All experimental protocols were approved by two ethical committees namely “Comitato Etico Indipendente dell ‘Azienda Ospedaliero-Universitaria di Bologna, Policlinico S. Orsola-Malpighi (Bologna, Italy)” and “comité consultatif de protection des personnes dans la recherche biomédicale, Le centre de lutte contre le cancer Léon-Bérard (Lyon, France)”. Sample collection was carried out in accordance with relevant guidelines and regulations of these two committees. As per guidelines of these two committees, we obtained informed consents of patients for use of their blood samples and the DNA extraction from bloods.

### Whole-exome/genome sequencing, mapping, variant calling, filtering and annotation

WES/WGS of the cases and controls from different families considered into the current study was performed in the Illumina X10 platform using DNA extracted from the blood samples. WES/WGS was performed as a paired-end sequencing with a read length of 150 bp. Mapping of reads to reference human genome (assembly version hs37d5) was performed using BWA-0.7.8.r2.05 mem (convey and alignment parameter as –T 0)^59^ and duplicates were removed using bammarkduplicates-0–0.148. Variants were called by using Platypus-0.8.1^13^ (with details as Platypus-0.8.1.py callVariants —genIndels = 1 —genSNPs = 1 —minFlank = 0). Variants were annotated using ANNOVAR^14^, dbNSFP v3.0^18^, 1000 Genomes phase III^17^, dbSNP^19^ and ExAC^15^. Variant filtering was performed with considering the quality score >20 and coverage of minimum 5 reads. Minor allele frequency (MAF) of 0.1% was used with respect to population databases (the 1000 Genomes phase III^17^, non-TCGA exome aggregation (ExAC) data^15^, and local data sets). A pairwise comparison of variants among the cohort was performed to check for sample swaps and family relatedness.

### Segregation in Pedigrees

The variants were filtered separately in each family based on the pedigree data by considering cancer patients as cases and unaffected persons as controls, and by applying to each individual a probability consideration for being a Mendelian case or a true control. However, a rule of thumb was that a maximum number of cases and a minimum number of controls in each family must carry the variant.

### Variant ranking using combined annotation dependent depletion (CADD)

After pedigree segregation, variants were prioritized using the CADD tool v1.3^20^ with the scaled PHRED-like CADD score greater than 10, which accounts for top 10% of probable deleterious variants in the human genome. Similarly the scores >20 and >30 are indicative of the top 1% and top 0.1% of deleterious variants, respectively^20^. All variants with CADD score >10 were taken into further consideration.

### Screening genic variants using intolerance score

Intolerance score ranks genes based on their capabilities to possess more or less common functional genetic variation relative to the genome wide expectation. Hence, genes, which have less common functional variation, are referred to as “intolerant” genes while genes prone to have more variations are called as “tolerant” genes. Tools were developed for calculating intolerance scores as reported in Table 1. Initially, residual variation intolerance score (RVIS) was developed based on allele frequency data as represented in whole exome sequence data from the NHLBI-ESP6500 data set^60^. We used this scoring system with ExAC^15^ and with our local datasets to create two additional intolerance scores, which were included into the prioritization process (Table 1).

Based on large-scale exome sequencing data, the ExAC consortium has developed two scoring systems which are known as loss-of-function (LoF) intolerance score (pLI score) and Z-score for missense and synonymous variants^15^. The pLI score is the probability that a gene is intolerant to a LoF mutation. There are three major classes for LoF mutations: (i) null, where LoF variation is completely tolerated; (ii) recessive, where heterozygous LoFs are tolerated; and (iii) haploinsufficient, where heterozygous LoFs are not tolerated. The closer the pLI score is to 1, the less tolerant the gene is to LoF, with pLI > = 0.9 reflecting an extremely LoF-intolerant set of genes. Similarly, the Z-score was developed by the ExAC consortium for missense and synonymous variants. The Z-score is based on the deviation of the observed from the expected number (Table 1). Positive Z-scores indicate that the gene has less variants than expected and hence is intolerant to variation, while genes with more variants will have negative Z scores^15^.

### Conservational screening of variants

Since high evolutionary conservation indicates functional importance of a position, it can be used to predict if a variant is deleterious or not. Based on this approach, evolutionary conservation-based parameters were developed as summarized in Table 1. Genomic Evolutionary Rate Profiling (GERP)^61^ and the PhastCons^62^ were utilized for the assessments of variant conservation with the GERP score of >2.0 and the PhastCons score of >0.3 indicating a high level of conservation of the variant position and were used as threshold in the screening of variants. PhyloP is a module of the PHAST package^63^, which calculates p-values for conservation using a defined multiple alignment^63^. PhyloP scores range from −14 to + 6 where a higher score indicates a higher level of conservation. During the variant ranking process, a PhyloP score ≥3.0 was used as a criterion for a high level of conservation (Table 1).

### Screening missense variants using 12 deleterious ranking tools

All missense variants were assessed for deleteriousness using 12 tools as summarized in Table 2. These tools were developed using information based on (a) sequence conservation, (b) structure, (c) combination of sequence and structure information and (d) meta-prediction using already known tools. SIFT is based on sequence data^64^, while PolyPhen uses both sequence and structure features^65^, and MetaSVM and MetaLR are combining pre-existing tools and hence these are examples of meta prediction tools^18^. Scores from these tools were gathered using dbNSFP^18^. Variants predicted to be deleterious by at least 60% of these tools were analysed further.

### Prediction of regulatory nature of the non-coding variants

Putative miRNA targets among the 3′ UTR variants were detected using the miRanda suite; a mirSVR score lower than −0.1 is indicative of a “good” miRNA target^7^. Furthermore, we used entire dataset of the human miRNA target atlas from targetscan 7.0^8^ and scanned it with help of the intersect function of the bedtools^66,67^. The 5′ UTRs were scanned for transcription factor binding sites using SNPnexus^9^. For regulatory variants, we merged enhancer^10^ and promoter^11,12^ data from the FANTOM5 consortium using the intersect function of bedtools. We employed a similar strategy for variants potentially localized in the super enhancer regions using super-enhancer archive (SEA)^25^ and dbSUPER^26^. Furthermore, the regulatory nature and impact of non-coding variants were assessed using CADD v1.3^20^, HaploReg V4^21^ and RegulomeDB^22^, which are based mainly on the ENCODE data^68^. SNPnexus^9^ was used to evaluate changes in transcription factor binding sites. Additionally, epigenomic data and marks from 127 cell lines from the NIH Roadmap Epigenomics Mapping Consortium were accessed via CADD v1.3^20^ for regulatory variants. We also tested if our variants were located within the ultra-conserved non-coding elements (UCNEs) and their clusters also known as ultra-conserved genomic regulatory blocks (UGRBs) with the help of UCNEbase^24^ and also if these variants were located in ultra-sensitive regions (Ultrasen), defined by FunSeq2^23^.

### Visualization of the variants

Variants were visualized in the human genome (version hg19) using the Locuszoom^27^, SNiPA^28^, the UCSC^29^ and ZENBU^30^ genome browsers.

### Ranked deleterious variants were examined for additional features

Potential variants were examined carefully for several additional features like if these variants were found in known list of CPGs^1^. It was also examined whether clinical data and associated phenotypic data from ClinVar^33^, Online Mendelian Inheritance in Man (OMIM, https://omim.org/) and other disease gene databases were available for the concerned variants. The status of RNA and protein expression for genes carrying potential deleterious variants was examined with the help of BioGPS^31^ and Human Protein Atlas^32^, respectively. We also checked if the concerned germline variant was already reported in known germline variant databases like CanVar Browser^34^ or in the somatic mutation cancer databases like cBioPortal^35^, COSMIC^36^, ICGC^37^ and IntOGen^38^. The sequencing data for the concerned variants were rechecked manually using Integrative Genomics Viewer (IGV)^39^ and validated using Sanger sequencing.

### Other data analysis by FCVPPv2

We can assist with data analysis using FCVPPv2, please send us a personal communication to either AK (a.kumar@dkfz.de) or AF (a.foersti@dkfz.de).

## Electronic supplementary material

Supplementary information 1

Supplementary table S2
