# Supplementary material for: Familial Cancer Variant Prioritization Pipeline version 2 (FCVPPv2) applied to a papillary thyroid cancer family
Source: Sci Rep. 2018 Aug 2;8:11635. doi: 10.1038/s41598-018-29952-z (PMC6072708; doi:10.1038/s41598-018-29952-z)
Supplement: Supplementary file 1 — Supplementary information 1 [file 41598_2018_29952_MOESM1_ESM.pdf]

Familial Cancer Variant Prioritization Pipeline version 2 (FCVPPv2) applied to a papillary thyroid cancer family

Abhishek Kumar[1,#], Obul Reddy Bandapalli[1,#], Nagarajan Paramasivam[2,3], Sara Giangiobbe[1], Chiara Diquigiovanni[4], Elena Bonora[4], Roland Eils[2,5], Matthias Schlesner[2,6], Kari Hemminki[1,7] and Asta Försti[1,7]

[1] Division of Molecular Genetic Epidemiology, German Cancer Research Center (DKFZ), D69120 Heidelberg, Germany;

[2] Division of Theoretical Bioinformatics, German Cancer Research Center (DKFZ), D69120 Heidelberg, Germany;

[3] Medical Faculty Heidelberg, Heidelberg University, D69120 Heidelberg, Germany;

[4] Unit of Medical Genetics, S.Orsola-Malpighi Hospital, 40138 Bologna, Italy;

[5] Department of Bioinformatics and Functional Genomics, Institute of Pharmacy and Molecular Biotechnology (IPMB) and BioQuant, Heidelberg University, D69120 Heidelberg, Germany;

[6] Bioinformatics and Omics Data Analytics, German Cancer Research Center (DKFZ), D69120 Heidelberg, Germany;

[7] Center for Primary Health Care Research, Lund University, Malmö, Sweden;

# Shared first and corresponding authorship

Key words: family-based, germline genetics, genetic risk factors, CPXM1, computational pipeline

Running title: FCVPPv2

Fig. S1. Location and sequencing summary of CPXM1 variant.

A. CPXM1 variant is located in the exon 11 of CPXM1 gene.

B. Visualization of the CPXM1 variant in genome sequencing data of the PTC family using the IGV browser. This variant is only found in two related cases (numbers 2 and 3) and absent in the unrelated case (number 1).

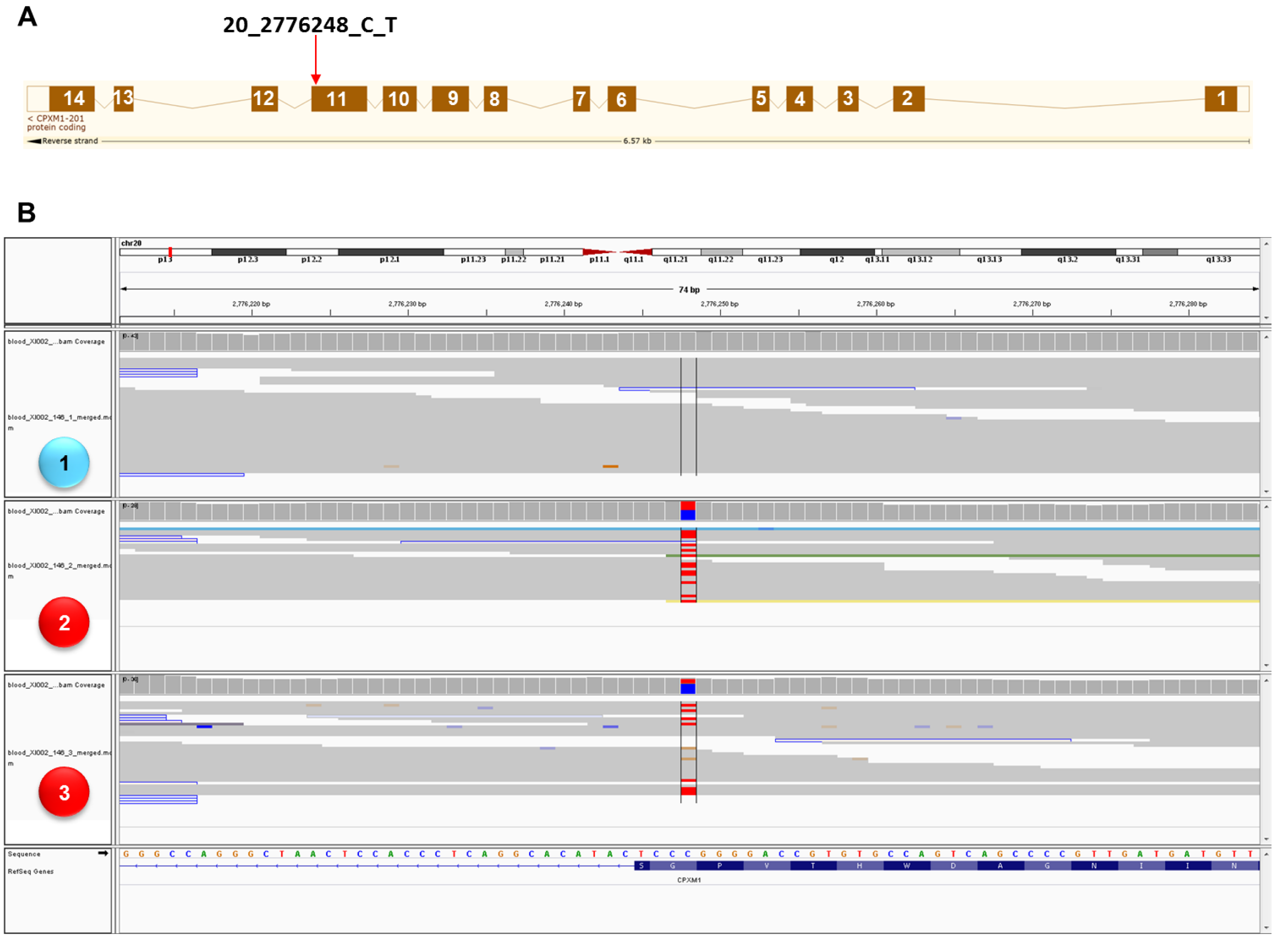

Supplementary table S1. List of abbreviations used in current study.

Abbreviations	Details
C1orf27	chromosome 1 open reading frame 27
CADD	Combined Annotation Dependent Depletion
CanVar	Cancer Variation Resource
COSMIC	Catalogue of Somatic Mutations in Cancer
CPGs	cancer predisposing genes
CPXM1	carboxypeptidase X (M14 family), member 1
dbSNP	database of Single Nucleotide Polymorphisms
dbSUPER	database of super enhancers
EVS6500	Exome Variant Server (EVS) with 6500 exomic samples (February, 2013)
ExAC	Exome Aggregation Consortium
FAM129A	family with sequence similarity 129, member A
FATHMM	Functional Analysis Through Hidden Markov Models
FCVPPv2	familial cancer variant prioritization pipeline
FCVPPv2	familial cancer variant prioritization pipeline version 2
GERP	Genomic Evolutionary Rate Profiling
ICGC	International Cancer Genome Consortium
IGV	Integrative Genomics Viewer
IntOGen	Integrative OncoGenomics
LRT	Log ratio test
LoF	loss of function
MAF	Minor allel frequency
MetaLR	Meta server based on logistic regression (LR)
MetaSVM	Meta server based on support vector machine (SVM)
miRNA	microRNA
NGS	Next Generation Sequencing
OMIM	Online Mendelian Inheritance in Man
PhyloP	Phylogenetic P-value
PolyPhen-v2	Polymorphism Phenotyping version 2
PROVEAN	Protein Variation Effect Analyzer
PTC	papillary thyroid cancer
QUAL	quality scores
RI	Reliability index
RVIS	Residual Variation Intolerance Score
SEA	super enhancer archive
SIFT	Sorting Intolerant from Tolerant
UCNEs	ultra conserved non-coding elements
UCSC	University of California, Santa Cruz
UGRBs	ultra conserved genomic regulatory blocks
Ultrasen	ultra sensitive regions

UTR	untranslated region
VEST3	Variant Effect Scoring Tool version 3.
WES	whole exome sequencing
WGS	whole genome sequencing
ZBTB41	zinc finger and BTB domain containing 41

Supplementary table S3. Overview of CPXM1 variants identified by whole-genome sequencing of members of 77 cancer families.

Family ID	VARIATION	Variants	AAchange	Cases*	Control**	CADD
CRC family 14	SNVs	20_2777048_C_T	E363K	0/2	1/1	33
CRC family 15	SNVs	20_2777864_G_A	A269V	1/2	0/2	22.2
HL family 1	SNVs	20_2777864_G_A	A269V	0/2	1/2	22.2
**PTC family**	**SNVs**	**20_2776248_C_T**	**G537R**	**2/2**	**0/0**	**32**

CRC – colorectal cancer, HL - Hodgkin's lymphoma and PTC - papillary thyroid cancer

* - No. of cases possessing variant/Total number of cases in the family

** - No. of controls possessing variant/Total number of controls in the family

Supplementary table S4. Overview of CPXM1 variants available in the Cancer Variation Resource for 1,006 familial early onset CRC patients via CanVar Browser.

Variant	SNPdb ID	Variant type	Aachange	Allele Count	Allele Numbers	Allele Frequency (AF)	ExAC_AF	CADD score
20_2776248_C_T	rs145736623	missense	G573R	1	1754	0.0005701	0.000364	32
20_2776527_G_A	rs41309351	missense	R480W	10	1568	0.006378	0.00222	15.4
20_2776975_C_T	rs41310169	missense	R387Q	17	1930	0.008808	0.00683	34
20_2777921_C_T	rs41301840	missense	R250H	4	1836	0.002179	0.00201	34
20_2778865_G_A	NA	stop gained	Q175*	1	1968	0.0005081	NA	35

CRC – colorectal cancer.
